# Adaptive Responses and Stress Coping Styles in Cardiac Surgery Patients: A Clinical Study

**DOI:** 10.3390/jcm15134909

**Published:** 2026-06-24

**Authors:** Grzegorz Wąchol, Martyna Tokarczyk

**Affiliations:** 1 Department of Philosophy, The Pontifical University of John Paul II in Krakow, 25 Kanonicza St., 31-002 Krakow, Poland; 2 Dr. J. Babinski Clinical Hospital in Krakow, 29 Babinskiego St., 30-393 Krakow, Poland; 3 Polish Psychologists’ Association (PPA), 1d, 238-246 King St., London W6 0RF, UK; m.tokarczyk.psycholog@gmail.com

**Keywords:** adaptive responses, stress coping styles, adaptation, cardiac surgery patient, heart surgery

## Abstract

**Background:** The present study examined coping styles and adaptive responses in patients undergoing cardiac surgery and healthy controls. **Methods:** A total of 240 participants were included, comprising cardiac surgery patients and a control group. The Coping Inventory for Stressful Situations and the Reactions to Impairment and Disability Inventory were used. **Results:** Significant group differences were found in adaptive responses, with cardiac surgery patients showing higher levels of shock, anxiety, denial, and overall maladaptive adjustment patterns. Patients also demonstrated a distinct profile of adaptation compared with healthy controls. Task-focused coping was associated with more adaptive functioning, whereas emotion-focused coping and maladaptive emotional responses were associated with poorer psychological adjustment. **Conclusions:** Overall, the results indicate that coping style is a key factor in psychological adaptation to cardiac surgery, with implications for the development of targeted psychological interventions in cardiac rehabilitation.

## 1. Introduction

Medical procedures are often considered among the most stressful experiences in an individual’s life. This is particularly true for cardiac surgeries, which are sometimes associated with high levels of psychological distress and uncertainty [1,2]. Patients awaiting cardiac surgery or interventions frequently experience fear, anxiety, and concerns regarding surgical outcomes. These feelings may negatively influence treatment adherence, emotional functioning, and postoperative recovery [2,3]. A considerable proportion of cardiac patients experience clinically significant symptoms of anxiety and depression [4]. At present, cardiovascular diseases remain one of the leading causes of morbidity and mortality worldwide, and their prevalence continues to increase due to population aging and growth [5].

Cardiac surgery is associated not only with psychological distress but also with pronounced physiological stress responses. Anticipation of surgery activates the hypothalamic–pituitary–adrenal axis and the sympathetic nervous system, resulting in increased cortisol secretion, inflammatory activation, and autonomic dysregulation. These physiological responses may adversely affect emotional functioning and are associated with slower postoperative recovery [4]. Consequently, individual differences in psychological regulation and stress management may play an important role in adaptation to undergoing cardiac surgery [6].

Coping refers to cognitive and behavioral efforts aimed at managing demands perceived as exceeding an individual’s personal resources [7]. According to Endler and Parker [8], three primary coping styles can be distinguished: task-focused coping, emotion-focused coping, and avoidance-focused coping. Task-focused coping is generally considered the most adaptive, as it is associated with lower perceived stress and better emotional regulation [9,10]. In cardiac populations, coping strategies have been linked to emotional well-being, quality of life, treatment adherence, and rehabilitation outcomes. In contrast, emotion-focused coping is more consistently associated with higher levels of psychological distress, including anxiety and depressive symptoms [11].

Psychological adaptation to illness involves a range of adaptive and maladaptive responses to disease-related stress and functional limitations [12]. Adaptive responses may include acceptance and constructive adjustment to illness, whereas maladaptive responses may involve anxiety, hostility, hopelessness, or depressive reactions. Some responses, such as denial, may temporarily reduce emotional distress during the early stages of illness. This may reflect the protective function of denial, which can help individuals cope with overwhelming information and emotional burden. However, persistent maladaptive responses, particularly depressive symptoms, are associated with poorer recovery, lower treatment adherence, and worse postoperative outcomes [13,14,15].

Although coping styles and psychological functioning have been widely investigated in chronic illness, relatively little is known about the relationships between coping styles and adaptive responses, specifically among patients undergoing cardiac surgery. Previous studies have often examined coping strategies or emotional outcomes separately rather than integrating these variables within a broader framework of psychological adaptation. A better understanding of these relationships may facilitate the identification of patients at risk of poorer psychological adjustment and support the development of targeted psychological interventions during cardiac rehabilitation.

The present study aimed to investigate the relationships between coping styles and adaptive responses among patients undergoing cardiac surgery, and to compare these variables with those observed in a healthy control group. We also examined which styles of coping with stress and other adaptive reactions are associated with adjustment (the most desirable reaction in the treatment process) and depression (understood not as a clinical disorder, but as a reaction that most hinders therapy).

Based on the analyses conducted so far, the following hypotheses were formulated:

**Hypothesis** **1.** *Patients undergoing cardiac surgery would demonstrate higher levels of maladaptive responses compared with healthy controls, after controlling for demographic differences.*

**Hypothesis** **2.** *Adjusment (the most desirable response in the treatment process) would be positively associated with task-focused coping and acceptance.*

**Hypothesis** **3.** *Depression (the reaction that most hinders the treatment process) would be positively associated with emotion-focused coping, shock and anxiety.*

## 2. Materials and Methods

### 2.1. Study Design

This study employed a single-center, cross-sectional observational design to examine coping styles and adaptive responses in patients undergoing cardiac surgery and in a healthy control group. Data collection was conducted in 2024 and 2025.

### 2.2. Participants

The study included a total of 240 participants (*N* = 240), divided into two equal groups: 120 cardiac patients (*N* = 120; 50%) and 120 healthy controls (*N* = 120; 50%).

The patient group consisted of individuals scheduled for cardiac surgery, including coronary artery bypass grafting, valve repair or replacement, aortic aneurysm surgery, and other cardiac procedures. The control group included individuals without a history of cardiovascular disease or cardiac surgery. Participants in this group reported no cardiac conditions and had no prior cardiac interventions.

The patient group was predominantly male (*N* = 96; 80.0%), with females accounting for 20.0% (*N* = 24). The mean age was 61.7 years (SD = 12.7; range: 23–77 years). The control group included 72 females (60.0%), 46 males (38.3%), and 2 participants identifying outside the binary gender classification (1.7%), with a mean age of 49 years (SD = 14.73; range: 18–73 years).

Inclusion criteria for the patient group were: (1) qualification for cardiac surgery, (2) age ≥ 18 years, and (3) ability to provide informed consent and complete the questionnaire independently. Exclusion criteria included: (1) diagnosed severe cognitive impairment or psychiatric disorders preventing questionnaire completion, and (2) incomplete questionnaire data.

Inclusion criteria for the control group were: (1) no history of cardiovascular disease or cardiac surgery, (2) age ≥ 18 years, and (3) ability to provide informed consent. Participants reporting cardiac conditions or previous cardiac interventions were excluded.

The groups were not matched for demographic characteristics. However, potential differences in age and gender were statistically controlled in the main analyses using covariates (age and gender) in regression models.

### 2.3. Procedure

Data collection was conducted in two stages. The first stage took place at the John Paul II Specialist Hospital in Kraków, where patients from the Department of Cardiac Surgery, Vascular Surgery, and Transplantology were recruited. Participants were informed about the aim of the study, ensuring anonymity and voluntary participation. Ethical approval was obtained from the Ethics Committee of the Pontifical University of John Paul II in Kraków (9 April 2024 no. KE/0104/2024/OP). The study was conducted in accordance with the Declaration of Helsinki, and informed consent was obtained from all participants.

The second stage involved recruitment of the control group. Participants were recruited voluntarily and completed the questionnaire in direct contact with the researcher. Respondents responded in the traditional way, completing paper forms. They were informed about the purpose of the study and assured confidentiality.

After data collection, all responses were coded and analyzed using IBM SPSS Statistics software (version: 29.0.2.0(20)).

### 2.4. Measures

In order to test the research hypotheses, a questionnaire survey was conducted, consisting of two parts. The first part included both closed and open-ended questions. These included: questions relating to the socio-demographic characteristics of the sample, e.g., questions about gender, age or education and original questions designed to gather specific information on previous operations and accidents. The second part of the study was based on two research tools: the CISS—Coping in Stressful Situations Questionnaire and the RIDI—Reactions to Impairment and Disability Inventory.

#### 2.4.1. Coping Inventory for Stressful Situations (CISS)

Coping styles were assessed using the Coping Inventory for Stressful Situations, in its Polish adaptation [7,8]. The questionnaire consists of 48 items rated on a 5-point Likert scale (1 = never to 5 = very often) and measures three coping styles: task-oriented (TASK), emotion-oriented (EMO), and avoidance-oriented coping (AVO). Avoidance coping includes two subdimensions: engaging in substitute activities and seeking social diversion. The Polish adaptation shows satisfactory psychometric properties. Reliability ranges from α = 0.74 to α = 0.85, depending on the scale.

#### 2.4.2. Reactions to Impairment and Disability Inventory (RIDI/KRP)

Psychological adaptation was measured using the Reactions to Impairment and Disability Inventory (RIDI), adapted into Polish as the Kwestionariusz Reakcji Przystosowawczych [16]. The instrument consists of 60 items rated on a 4-point Likert scale and assesses eight adaptation-related responses: shock, denial, anxiety, depression, internalized anger, externalized hostility, acceptance, and adjustment. The RIDI/KRP consists of eight independent subscales. Conceptually, acceptance and adjustment are regarded as adaptive responses, anxiety, depression, internalized anger, and externalized hostility as maladaptive responses, whereas denial represents a distinct denial-related reaction. However, this classification is theoretical and does not reflect computed higher-order or composite scores [12]. Therefore, all subscale scores were analyzed separately. The Polish adaptation demonstrates satisfactory psychometric properties and a stable factor structure, with reliability coefficients ranging from α = 0.78 to α = 0.88 depending on the subscale.

### 2.5. Statistical Analysis

The analyses were conducted using IBM SPSS Statistics. Independent-samples *t*-tests were applied. Since the data were not normally distributed, the non-parametric Mann–Whitney U test was initially used; however, its results were consistent with those obtained from the *t*-tests. Effect sizes of differences between the study and control groups were also measured using Cohen’s d coefficient. Relationships between variables were examined using correlation and regression analyses. Statistical significance was set at *p* < 0.05. For regression models, 95% confidence intervals were reported.

The power of the study was calculated. A post hoc test showed that for an expected effect of 0.4 and an assumed error of 0.05, a very high coefficient of (1 − β) = 0.99 was obtained for the correlational study. For the between-group study, with the same assumptions and comparison groups (*N* = 120), a (1 − β) = 0.87 was obtained. This is a satisfactory result.

## 3. Results

A comparative analysis of the study group (*N* = 120) and the control group (*N* = 120) revealed significant differences in the severity of four adaptive reactions.

The largest difference was observed in denial. Patients scored significantly higher on average (M = 17.25) than the control group (M = 13.33). A large effect size (d = 0.83) was also observed in this case, indicating that it is easily detectable and may have significant practical implications.

For adjustment reactions, patients scored higher on average (M = 23.20) than the control group (M = 19.93), and the effect size was moderate (d = 0.50).

For shock, patients scored slightly higher on average (M = 12.91) than the control group, and the effect size was also moderate (d = 0.48).

Also for anxiety reactions, patients scored slightly higher on average (M = 13.52) than the control group (11.55), with a moderate effect size (d = 0.48). The results are presented in Table 1.

Descriptive statistics for the three stress-coping styles were calculated for both groups. The task-focused coping style was predominant in both patients and controls, while the emotion-focused style had the lowest mean scores in both groups. Detailed statistics are presented in Table 2.

Relationships between coping styles and adaptive responses were then analyzed for patients only, using Pearson’s correlation coefficients, since the focus was on the treatment process. The control group was excluded from this analysis. The detailed results are shown in Table 3.

Hypothesis 2 was supported. Adjustment was significantly positively correlated with task-oriented coping, acceptance, and denial. All obtained results are positive and range from r = 0.30 to r = 0.44. In the next step, it was decided to test whether such a model could explain adjustment, so a regression analysis (enter method) was performed. The results for Hypothesis 2 are presented in Table 4.

The model explained 26% of the variance in adjustment and demonstrated good fit (F = 7.854; *p* < 0.001; Cl = 95%). Although task-focused coping was not a significant predictor within the model, removing it reduced the explained variance by less than 1%, indicating that denial and acceptance are the strongest predictors of adjustment. Univariate regressions for each predictor separately showed that all were statistically significant.

Hypothesis 3 was supported. Depression was significantly associated with emotion-focused coping, shock, anxiety and additionally with internal anger and external hostility. All obtained results are positive and range from r = 0.22 to r = 0.67. In the next step, it was decided to test whether such a model could explain depression, so a regression analysis (enter method) was performed. Results related to Hypothesis 3 are presented in Table 5.

This regression model explained 55% of the variance in depression and showed a good fit (F = 15.408; *p* < 0.001; Cl = 95%). Among the predictors, only shock was statistically significant; however, removing the other variables led to a decrease of over 7% in explained variance (R^2^ dropped to 0.479). Single-factor regression analyses indicated that all predictors were individually significant.

## 4. Discussion

Findings suggest differences in adaptive responses between cardiac surgery patients and the control group, particularly in shock, anxiety, denial, and adjustment. These reactions are typically observed in acute health-related crises. Such responses may reflect the initial phase of psychological adaptation to serious or life-threatening illness [5,17,18,19].

The results suggest the importance of providing psychological support during the early stages of hospitalization, and possibly even before admission. Overly formal clinician–patient communication may increase anxiety and hinder a supportive therapeutic environment. Both acceptance and temporary emotional distancing appear to contribute to adaptation to illness [20,21].

Other adaptive reactions, such as depression, internalized anger, externalized hostility, and acceptance, did not differ significantly between groups. These reactions appear to be characteristic of acute health-related crises such as cardiac surgery. Other reactions may be more common across everyday stress contexts [15].

Moreover, findings suggest associations between adaptive responses and task-focused coping, as well as denial and acceptance. Task-focused coping was associated with more adaptive functioning, as it facilitates active engagement and problem-solving in stressful situations. Limited acceptance may hinder engagement in adaptive coping, whereas excessive focus on negative outcomes may contribute to cognitive overload and reduced adaptability [5].

This may suggest that adaptation involves a balance between acceptance of illness and temporary psychological distancing from distressing information. Task-focused coping may support preparation for surgery and engagement in rehabilitation, while psychological support may reduce excessive focus on distressing anticipatory thoughts and information overload. At the same time, acceptance of one’s limitations remains an important component of adjustment.

Results are consistent with previous studies highlighting the effectiveness of task-focused strategies under stress [22]. Research by Eysenck et al., Vickers, and Oudejans indicates that interventions aimed at improving attentional control may be effective in high-stress performance contexts [23,24,25]. Such interventions may be delivered not only by psychologists but also by trained medical staff.

Positive associations were also found between depressive reactions and emotion-focused coping, as well as shock, anxiety, internalized anger, and externalized hostility. Emotion-focused coping is associated with increased emotional and somatic distress. Previous research links this coping style to poorer psychological outcomes [26,27].

Shock reflects an acute emotional reaction to unexpected and overwhelming situations [5,18]. Anxiety may impair coping processes [28]. However, internalized anger and externalized hostility reduce perceived control and difficulties in emotion regulation [29,30].

Emotion-focused coping and related reactions contribute to depressive responses, although only shock reached statistical significance. Hostile reactions may also be associated with emotional regulation difficulties, while externalized hostility reflects an external locus of control. Patient aggression in medical settings has been previously documented and is a complex phenomenon influenced by both situational and psychological factors [31,32].

Research also indicates that a common cause of patient dissatisfaction and aggression is an overly formal approach, the use of incomprehensible medical language, and a lack of personal approach by staff.

Findings highlight the importance of emotional regulation and psychological support in cardiac patients. Interventions such as emotional awareness training and mentalization-based approaches may help reduce distress and improve patient–staff communication.

Further research should examine the role of individual differences in adaptive responses, particularly in relation to personality traits, emotional regulation capacities (including emotional intelligence), cognitive functioning, and attentional control. Such studies would allow for a more comprehensive understanding of adaptive processes in medically induced stress conditions and could contribute to identifying psychological profiles associated with more effective adaptation to cardiac surgery and rehabilitation.

The next step in further research, based on the obtained results, could be to develop training programs for medical personnel that would facilitate psychological support. Many elements of this support can be provided by doctors, nurses, and medical assistants during routine preoperative activities and during recovery. Furthermore, it seems crucial to systematically develop frameworks and models for specific psychological interventions for patients. These should encourage more focused activity and safe emotional expression. In the case of serious medical procedures, psychological care is an essential part of the entire therapeutic process. Numerous studies highlight the link between the quality of somatic treatment and mental well-being [33,34].

## 5. Limitations

The study is limited by potential sample-related constraints. Although the sample size was adequate for the analyses conducted, it may still have limited statistical power in some regression models. Differences in age and gender between groups may have introduced confounding effects despite statistical control. The heterogeneity of cardiovascular diagnoses is another limitation, as different clinical conditions may influence psychological responses.

## 6. Conclusions

The present findings suggest that psychological adaptation in cardiac surgery patients is strongly influenced by coping styles. Task-focused coping appears to support adaptive functioning, whereas emotion-focused coping is associated with greater psychological distress. Psychological interventions should focus on strengthening self-regulation, promoting adaptive coping strategies, and facilitating emotional expression. These approaches may improve adjustment during both pre- and post-surgical phases.

## Figures and Tables

**Table 1:** Mean scores of adaptive responses among patients and the control group and their differences.

	M CG	M P	Range	Diff (CG-P)	*t*	df	*p*	d Coehn’s
**shock**	**11.28**	**12.91**	**7–28**	**−1.63**	**2.25**	**118**	**0.026**	**0.41**
**anxiety**	**11.55**	**13.52**	**8–32**	**−1.97**	**2.64**	**118**	**0.009**	**0.48**
**denial**	**13.33**	**17.25**	**7–28**	**−3.92**	**4.58**	**118**	**<0.001**	**0.83**
depression	12.87	13.38	8–32	−0.51	0.65	118	0.259	0.12
inner anger	13.10	12.53	8–32	0.57	−0.71	118	0.241	−0.13
external hostility	12.38	12.03	7–28	0.35	−0.51	118	0.305	−0.09
acceptance	15.65	16.08	7–28	−0.43	0.51	118	0.306	0.09
**adjusment**	**19.93**	**23.20**	**8–32**	**−3.27**	**2.74**	**118**	**0.004**	**0.50**

M—mean; CG—control group; P—patients; diff—different; *t*—result of *t*-test; df—freedom degree; *p*—significance; bold—statistically significant result.

**Table 2:** Styles of coping with stress.

	Style	*N*	Min	Max	SD	M	Range
patients	task	120	29.00	75.00	8.97	52.43	16–80
	avoidance	120	24.00	66.00	8.71	43.85	16–80
	emotional	120	20.00	57.00	10.35	38.30	16–80
control group	task	120	32.00	75.00	10.41	55.37	16–80
	avoidance	120	16.00	66.00	11.36	43.05	16–80
	emotional	120	16.00	68.00	10.43	41.37	16–80

*N*—number of respondents; Min—minimum result; Max—maximum result; SD—standard deviation; M—mean.

**Table 3:** Pearson correlations between stress coping styles and adaptive responses.

		TASK	AVO	EMO	SCHO	ANX	DEN	DEPR	IANG	EHOS	ACCE
stress coping style	AVO	0.017									
						
	EMO	0.187	0.426 **								
adaptive responses	SCHO	−0.016	0.168	0.461 **							
	ANX	−0.068	0.108	0.420 **	0.587 **						
	DEN	0.089	0.232	0.177	0.299 *	0.267 *					
	DEPR	−0.072	0.154	0.448 **	0.698 **	0.562 **	0.185				
	IANG	−0.018	0.050	0.374 **	0.766 **	0.541 **	0.149	0.671 **			
	EHOS	0.171	0.235	0.347 **	0.464 **	0.420 **	0.228	0.545 **	0.586 **		
	ACCE	0.231	0.215	0.238	0.225	0.183	0.348 **	0.218	0.178	0.286 *	
	ADJU	0.300 *	−0.026	−0.145	−0.093	−0.089	0.390 **	−0.026	−0.093	0.107	0.440 **
−1.0 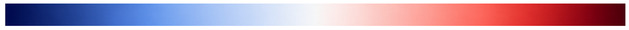 1.0											

TASK—task-focused coping style; AVO—avoidance-focused coping style; EMO—emotion-focused coping style; SCHO—shock; ANX—anxiety; DEN—denial; DEPR—depression; IANG—inner anger; EHOS—external hostility; ACCE—acceptance; ADJU—adjustment; *—correlation is significant at the 0.05 level (2-tailed); **—Correlation is significant at the 0.01 level (2-tailed).

**Table 4:** Regression model explaining adjustment by task-focused coping style, denial, and acceptance.

	B	SE	β	*p*
*F* (3;116) = 7.85; *p* < 0.001; *R^2^*_adj._ = 0.26				
task	0.125	0.071	0.203	0.084
denial	0.353	0.158	0.267	0.029
acceptance	0.396	0.161	0.300	0.017

Note: Dependent variable: Adjustment. Multiple Linear Regression. Enter method.

**Table 5:** Regression model explaining depression by emotion-focused coping style, shock, anxiety, inner anger, and external hostility.

	B	SE	β	*p*
*F* (5;114) = 15.41; *p* < 0.001; *R^2^*_adj._ = 0.55				
emotional	0.037	0.038	0.097	0.343
shock	0.326	0.142	0.338	0.026
anxiety	0.130	0.104	0.141	0.217
inner anger	0.190	0.148	0.193	0.204
external hostility	0.207	0.125	0.183	0.103

Note: Dependent variable: Depression. Multiple Linear Regression. Enter method.

## Data Availability

The original contributions presented in this study are included in the article. Further inquiries can be directed to the corresponding authors.
